# Pyrolysis Temperature-Dependent Biochar Regulates Lead Immobilization and Antioxidant Defense in *Wedelia trilobata* for Contaminated Soil Remediation

**DOI:** 10.3390/plants15213445

**Published:** 2026-11-05

**Authors:** Adeel Abbas, Rashida Hameed, Sidra Balooch, Shimeng Yang, Aitezaz Ali Shahani, Usman Zulfiqar, Mohammed Zari

**Affiliations:** 1 Biomedical Engineering and Bioengineering Laboratory, School of Health Management, Qingdao Hengxing University of Science and Technology, Qingdao 266100, China; adeel.abbas92@ujs.edu.cn (A.A.);; 2 Key Laboratory of Marine Chemistry Theory and Technology, Ministry of Education, College of Chemistry and Chemical Engineering, Ocean University of China, Qingdao 266100, China; 3 Institute of Urban Agriculture, Chinese Academy of Agricultural Sciences, Chengdu National Agricultural Science and Technology Center, Chengdu 610213, China; sidra1151214@gmail.com; 4 Laboratory of Crop Science, Agriculture College, Yanbian University, Yanji 133002, China; 2193609022@ybu.edu.cn; 5 Department of Agronomy, Faculty of Agriculture and Environment, The Islamia University of Bahawalpur, Bahawalpur 63100, Pakistan; usman.zulfiqar@iub.edu.pk; 6 Department of Biology, Nakhchivan State University, AZ7012 Nakhchivan, Azerbaijan; 7 Department of Environment, Faculty of Environmental Sciences, King Abdulaziz University, P.O. Box 80200, Jeddah 21589, Saudi Arabia

**Keywords:** rice straw biochar, lead contamination, soil remediation, *Wedelia trilobata*, antioxidant defense, pyrolysis temperature

## Abstract

This study investigated the effects of rice straw biochar produced at 450 °C (RBC450) and 700 °C (RBC700) on Pb immobilization, soil properties, and the physiological and molecular responses of *Wedelia trilobata* (L.) grown in Pb-contaminated soil. Biochar was applied at 1%, 2.5%, and 5% (*w*/*w*) to soil contaminated with 150 mg kg^−1^ Pb. Changes in soil physicochemical properties, Pb availability and accumulation in plant tissues, physiological performance, oxidative stress indicators, antioxidant enzyme activities, and antioxidant-related gene expression were evaluated. Both biochars reduced soil-available Pb and Pb accumulation in roots and shoots, with RBC700 showing greater Pb immobilization. Biochar application also improved soil pH, electrical conductivity, organic carbon, nitrate nitrogen, and available phosphorus. In plants, biochar enhanced catalase and peroxidase activities while reducing malondialdehyde accumulation and reactive oxygen species production, indicating alleviation of Pb-induced oxidative stress. qRT-PCR analysis further showed modulation of SOD-, POD-, and CAT-related antioxidant responses under Pb stress, with RBC450 treatment strengthening antioxidant defense at the transcriptional level. Overall, rice straw biochar reduced soil-extractable Pb and modified soil physicochemical properties and antioxidant responses in *W. trilobata*, with RBC700 showing greater effectiveness in decreasing Pb extractability. However, these changes did not consistently restore all physiological parameters under Pb stress.

## 1. Introduction

Lead (Pb) contamination in agricultural soils is a widespread and persistent environmental problem caused by rapid industrialization, mining activities, excessive use of agrochemicals, wastewater irrigation, and urban deposition [1]. Pb is a non-essential and highly toxic heavy metal with no known biological function in plants, animals, or microorganisms [2]. Even at low concentrations, Pb can persist in soil for decades due to its strong affinity for organic matter and clay minerals, making it highly stable and difficult to remove through natural processes [3]. Once accumulated in soils, Pb disrupts soil microbial communities, reduces enzyme activity, and interferes with nutrient cycling, ultimately degrading soil fertility and ecosystem productivity [4]. In plants, Pb toxicity manifests through multiple physiological and biochemical disturbances, including inhibition of seed germination, reduction in root elongation, disruption of chlorophyll synthesis, impairment of photosynthetic electron transport, and decreased nutrient uptake efficiency [5,6]. One of the most critical consequences of Pb stress is the overproduction of reactive oxygen species (ROS), which leads to oxidative damage of lipids, proteins, and nucleic acids [7]. This oxidative stress is commonly reflected by increased levels of malondialdehyde (MDA) and altered activity of antioxidant enzymes such as superoxide dismutase (SOD), peroxidase (POD), and catalase (CAT) [8]. Therefore, understanding both physiological and molecular responses is essential for evaluating plant tolerance under Pb stress conditions [9]. Traditional remediation approaches such as excavation, soil washing, and chemical stabilization are often expensive, labor-intensive, and environmentally disruptive, making them unsuitable for large-scale soil restoration [10]. In contrast, in situ remediation strategies using amendments such as biochar and engineered nanomaterials have gained increasing attention due to their efficiency, cost-effectiveness, and environmental compatibility [11]. Nanomaterials offer high surface reactivity and strong adsorption capacity for heavy metals; however, concerns remain regarding their long-term ecological risks, mobility in soil systems, and potential toxicity to non-target organisms [12]. These limitations have encouraged the exploration of more stable and sustainable soil amendments. Biochar, a carbon-rich material produced through pyrolysis of biomass under limited oxygen conditions, has emerged as a promising soil amendment for heavy metal remediation and soil quality improvement. Its physicochemical properties including high porosity, aromatic carbon structure, and diverse oxygen-containing functional groups (–COOH, –OH, and C=O) enable strong adsorption and immobilization of heavy metals such as Pb through electrostatic attraction, ion exchange, surface complexation, and precipitation mechanisms [13]. In addition, biochar may alleviate soil acidity, enhance cation exchange capacity (CEC), improve water retention, and influence nutrient availability. However, excessive increases in soil pH may negatively affect nutrient availability and plant growth, particularly in initially neutral or alkaline soils.

Importantly, biochar not only reduces the bioavailability of Pb in soil but also limits its uptake and translocation within plants, thereby reducing phytotoxic effects [14]. It also contributes to the reduction in ROS generation by lowering metal-induced oxidative stress, indirectly improving plant metabolic stability and stress tolerance [15]. Recent studies have further highlighted that pyrolysis temperature significantly influences biochar performance, with high-temperature biochar typically exhibiting greater aromaticity, stability, and adsorption capacity compared to low-temperature biochar [16]. However, comparative studies evaluating different pyrolysis temperatures under Pb stress conditions remain limited. *W. trilobata* is a fast-growing perennial plant species known for its high biomass production, strong adaptability, and potential application in phytoremediation systems. Despite these advantages, Pb toxicity significantly restricts its physiological performance by damaging cellular structures, disrupting enzymatic functions, and suppressing growth-related metabolic pathways [17]. These limitations reduce its effectiveness in heavily contaminated soils, highlighting the need for supportive amendments to enhance its remediation capacity. Recent research suggests that combining biochar with plant-based remediation systems can significantly enhance heavy metal immobilization and improve plant stress tolerance compared to single remediation approaches [18]. However, most existing studies focus primarily on soil chemical properties and plant physiological responses, while the underlying molecular mechanisms particularly antioxidant gene regulation remain insufficiently explored. At the molecular level, antioxidant defense genes such as SOD, POD, and CAT play a central role in scavenging ROS and maintaining cellular redox balance under heavy metal stress [19]. The expression of these genes reflects the activation of plant defense mechanisms and provides insight into stress adaptation at the transcriptional level [20]. Therefore, integrating physiological, biochemical, soil chemical, and molecular gene expression analyses offers a more comprehensive understanding of plant–soil–biochar interactions under Pb stress. In this context, the present study evaluates the effects of rice straw-derived biochar produced at 450 °C and 700 °C on Pb-contaminated soil and *W. trilobata*. The specific objectives of this study are to (i) assess the efficiency of biochar in immobilizing Pb and improving soil physicochemical properties, (ii) evaluate its impact on plant growth, physiological performance, and oxidative stress indicators, and (iii) investigate the transcriptional regulation of antioxidant genes (SOD, POD, CAT) using qRT-PCR. This study provides integrated soil–plant–molecular evidence for understanding biochar-mediated Pb stress alleviation and contributes to the development of sustainable and eco-friendly remediation strategies for contaminated agricultural soils.

## 2. Results

### 2.1. Biochar Properties

**Figure 1:**
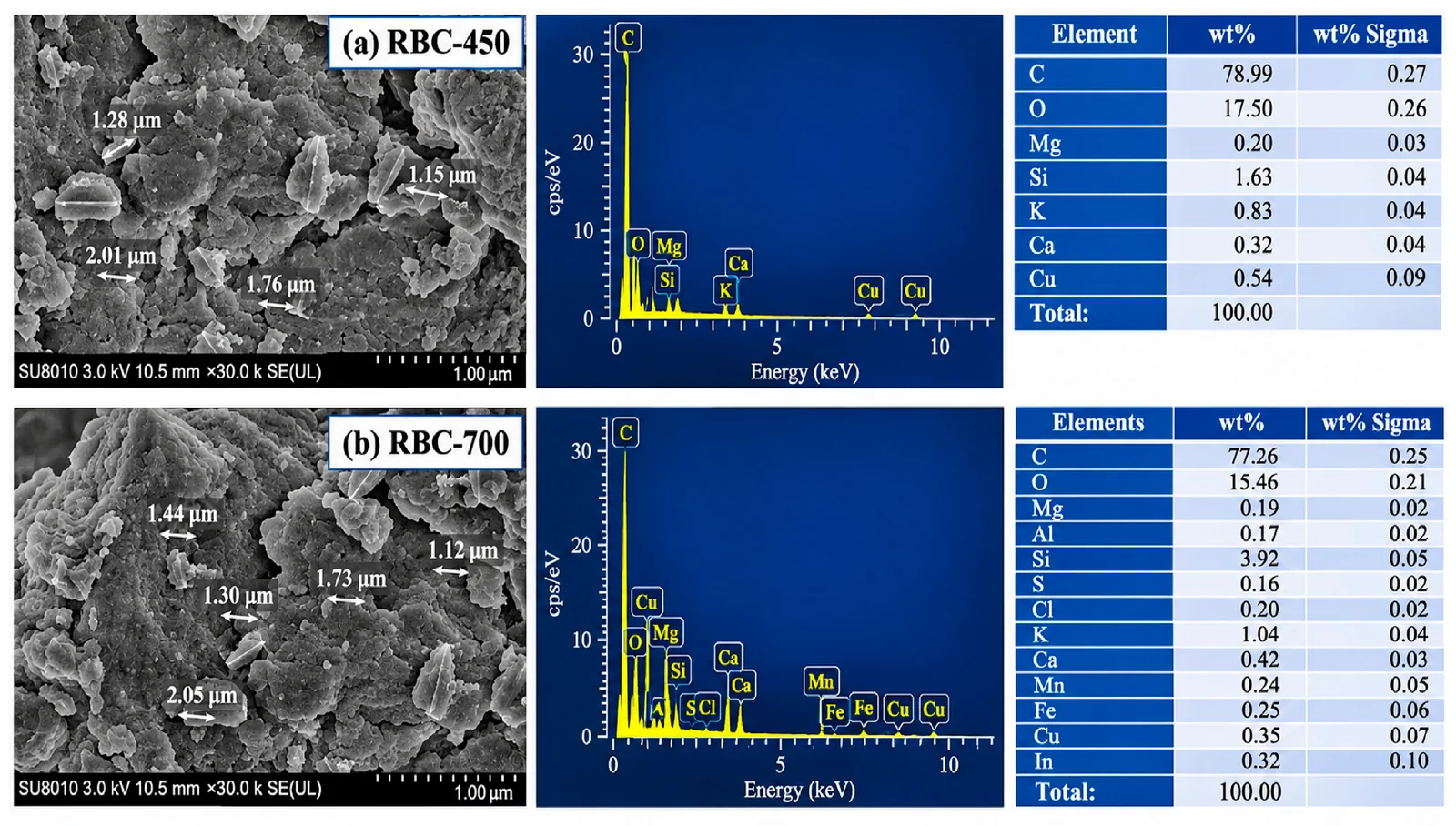
Scanning electron microscopy (SEM) images and energy-dispersive X-ray spectroscopy (EDX) elemental profiles of rice straw biochar produced at 450 °C (RBC450) and 700 °C (RBC700).

The SEM micrographs of RBC450 and RBC700 revealed irregular particle morphology and heterogeneous surface features (Figure 1). Although differences in surface morphology were observed, distinct pore structures could not be conclusively identified from the SEM images. Ball milling resulted in a heterogeneous distribution of particle sizes in both biochars. EDX analysis indicated that carbon was the predominant detected element in both RBC450 and RBC700, with variations in their elemental compositions (Figure 1). However, because EDX analysis cannot identify specific surface functional groups, no conclusions regarding the formation of oxygen-containing functional groups were drawn from these observations. The physicochemical characteristics of RBC450 and RBC700 are summarized in Supplementary Table S1. RBC700 exhibited a higher reported specific surface area (195.2 m^2^ g^−1^) than RBC450 (22.14 m^2^ g^−1^). Similarly, biochar pH increased from 7.52 to 9.61, while ash content increased from 17.64% to 25.63% with increasing pyrolysis temperature. In contrast, dissolved organic carbon (DOC) decreased from 8.32 mg C L^−1^ in RBC450 to 2.14 mg C L^−1^ in RBC700. These observations indicate that pyrolysis temperature influenced the reported physicochemical characteristics of rice straw biochar.

### 2.2. Improvement of Soil pH, EC, OC and Nutrients

Table 1 and Table 2 displays the soil pH, EC, and OC for Pb-contaminated soils amended with RBC450 and RBC700. The initial soil pH stood at 6.74, while amendments with RBC450 and RBC700 markedly elevated the pH levels. Specifically, RBC700 notably raised the soil pH to 7.76, whereas RBC450 slightly reduced it to 7.68. In terms of EC, all soils amended with RBC showed a significant compared with the Pb-free control (CN) treatment. RBC450 exhibited the highest EC value (0.67 dsm^−1^), with the rate of increase correlating with the amount of RBC added. Notably, the 5%RBC450 treatment showed the largest increase in EC compared to CN and RBC700 treatments (Table 2). After 60 days, soil NH_4_^+^-N concentrations remained approximately 40 mg kg^−1^ in the unamended control treatments, whereas biochar-amended treatments showed lower concentrations, ranging from 4.63 to 9.24 mg kg^−1^ (Table 2). These results indicate that biochar amendment was associated with a reduction in soil NH_4_^+^-N concentrations, although the magnitude of the response varied among treatments. In contrast, NO_3_^−^-N content in soils amended with RBC was elevated compared to the control soil, with a significant rise observed alongside increasing RBC addition rates. The most pronounced increase was noted in the 5%RBC700 treatment, surpassing levels in the CN treatment. The impact of varying RBC application rates on organic carbon was substantial (Table 2). There was a significant uptick in OC content with the escalation of RBC addition rates, where the OC content in the 5%RBC700 treatment exceeded that of the CN and RBC450 treatments. Soil OC increased 27.5 and 30.5 g kg^−1^, respectively, as RBC450 and RBC700 were employed, compared to control soil (4.58 g kg^−1^). Available phosphorus (AP) also increased following biochar amendment, with reported concentrations of approximately 29.5–29.6 mg kg^−1^ in the unamended controls and 59.3–63.6 mg kg^−1^ in selected 5% biochar treatments after 60 days (Table 2).

### 2.3. Available Pb in Soil

As shown in Figure 4d, DTPA-extractable Pb concentrations decreased in Pb-contaminated soils following rice straw biochar amendment. This reduction varied with biochar pyrolysis temperature and application rate. Specifically, available Pb concentrations were 50.5, 46.8, and 35.2 mg kg^−1^ in the RBC450 treatments and 49.7, 40.6, and 26.4 mg kg^−1^ in the RBC700 treatments at biochar application rates of 1%, 2.5%, and 5%, respectively (Figure 4d). These results indicate that increasing biochar application rates reduced Pb availability in the contaminated soil. The lower available Pb concentrations observed with RBC700, particularly at the higher application rates, indicate greater Pb immobilization compared with RBC450.

### 2.4. Pb Accumulation in Plant Roots and Shoots

**Figure 2:**
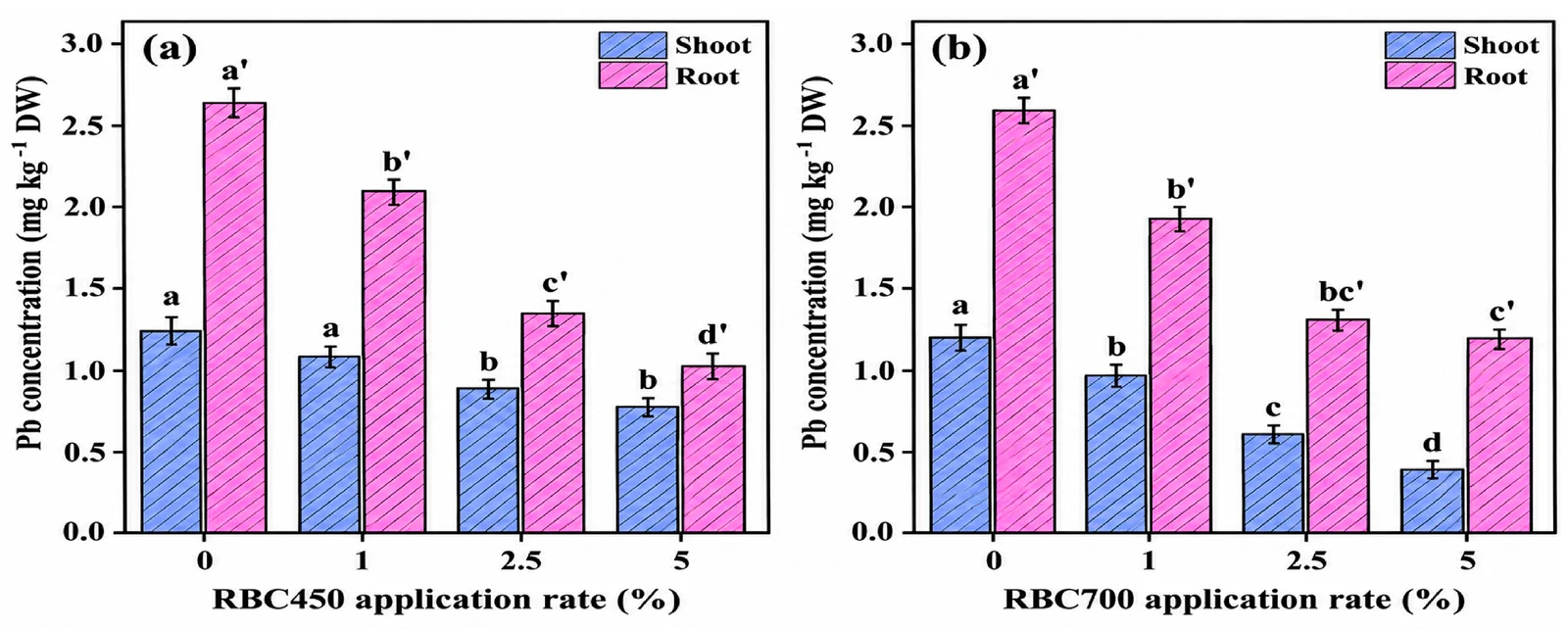
Effects of rice straw biochar produced at (**a**) 450 °C (RBC450) and (**b**) 700 °C (RBC700) on Pb concentrations in the shoots and roots of Wedelia trilobata under Pb contamination (150 mg kg^−1^ soil). Pb represents the contaminated control without biochar, while Pb-RBC450-1, Pb-RBC450-2.5, Pb-RBC450-5, Pb-RBC700-1, Pb-RBC700-2.5, and Pb-RBC700-5 represent the respective biochar treatments at 1%, 2.5%, and 5% (*w*/*w*). Different lowercase letters indicate significant differences among treatments within each plant tissue (*p* < 0.05).

Biochar had a notable impact on the concentration of Pb in plant compared to the control, as depicted in Figure 2. Analysis of Pb partitioning between roots and shoots revealed that Pb concentrations remained higher in roots than in respective shoots. Specifically, the concentration of Pb in shoots decreased with 1%, 2.5%, and 5.0% biochar amendments, respectively, compared to the control. Additionally, RBC700 showed higher reductions in Pb concentrations in roots, compared to the RBC450 and CN.

### 2.5. Effects of Biochar on Plant Physiological Responses and Antioxidant Enzyme Activities Under Pb Stress

**Figure 3:**
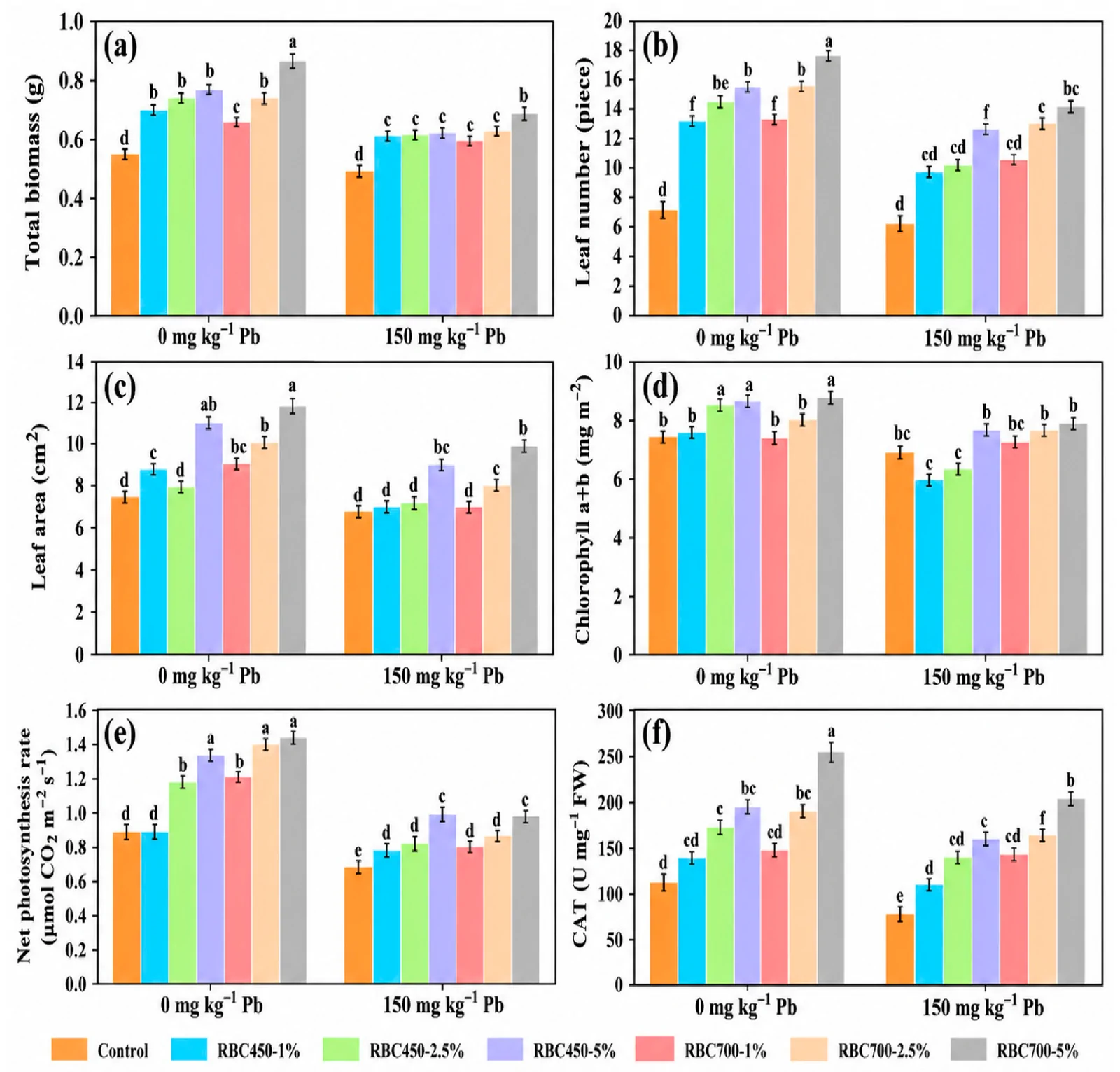
Effects of rice straw biochar (RBC450 and RBC700) on (**a**) total biomass, (**b**) leaf number, (**c**) leaf area, (**d**) chlorophyll a + b content, (**e**) net photosynthetic rate, and (**f**) catalase (CAT) activity in *W. trilobata* under Pb-free (0 mg kg^−1^) and Pb-contaminated (150 mg kg^−1^) conditions. Bar colors represent the control (0% biochar) and RBC450 or RBC700 applied at 1%, 2.5%, and 5% (*w*/*w*), as indicated in the legend. Error bars represent SE (n = 3 independent pots). Different lowercase letters indicate significant differences among treatments (*p* < 0.05).

**Figure 4:**
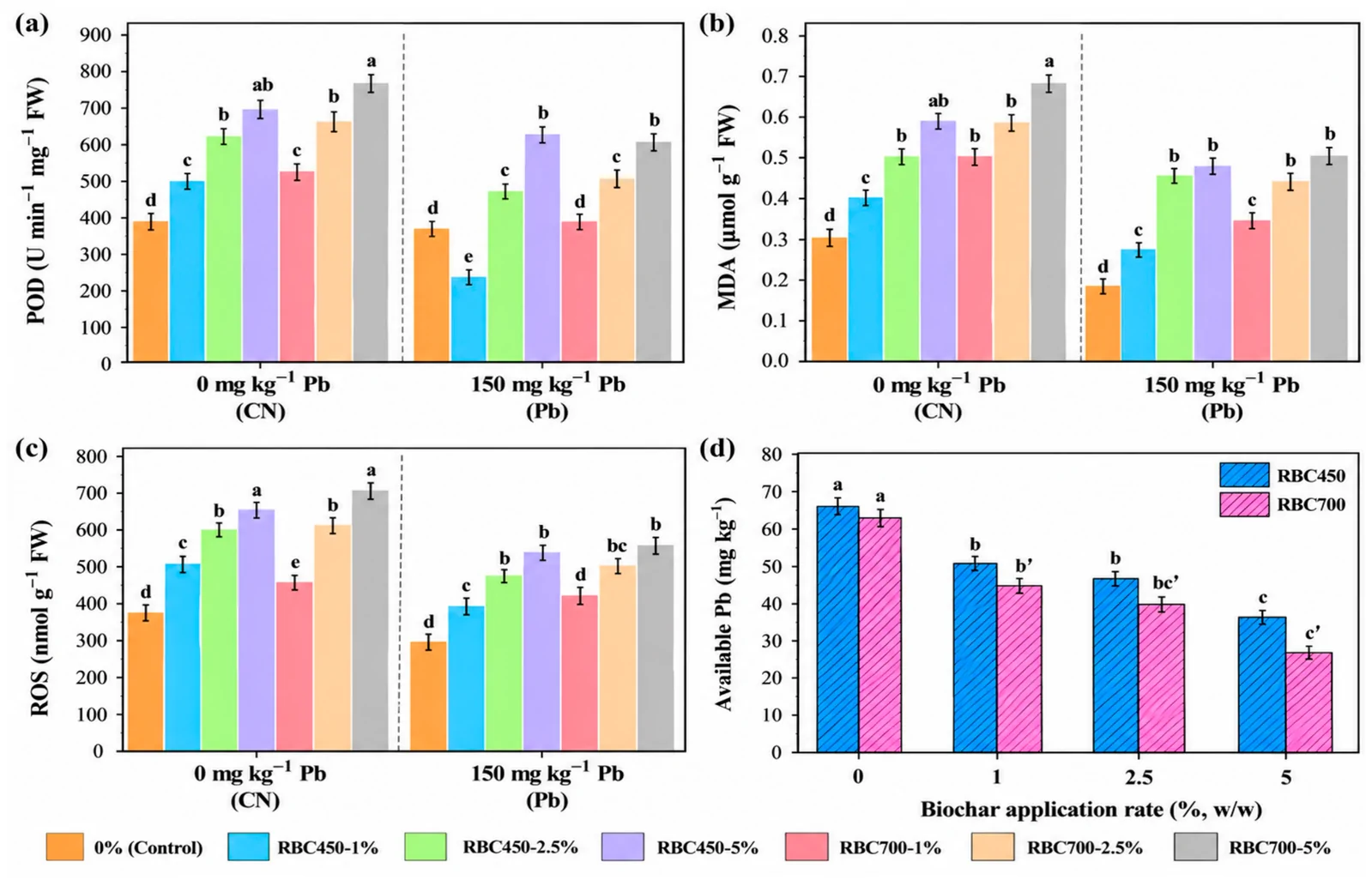
Effects of rice straw biochar (RBC450 and RBC700) on (**a**) peroxidase (POD) activity, (**b**) malondialdehyde (MDA) content, and (**c**) reactive oxygen species (ROS) levels in *W. trilobata* under Pb-free (0 mg kg^−1^) and Pb-contaminated (150 mg kg^−1^) conditions, and (**d**) soil DTPA-extractable Pb concentrations under Pb contamination. Bar colors represent the control (0% biochar) and RBC450 or RBC700 applied at 1%, 2.5%, and 5% (*w*/*w*), as indicated in the legends. Error bars represent SE (n = 3 independent pots). Different lowercase letters indicate significant differences among treatments (*p* < 0.05).

Figure 3 presents the physiological and biochemical responses of *W. trilobata* to different application rates of RBC450 and RBC700 under Pb-free and Pb-contaminated soil conditions. CAT and POD activities demonstrated differential activation profiles, exhibiting enhanced and elevated values at biochar incorporations of RBC450 and RBC700 in lead-polluted soil, relative to the CN depicted in the referenced figure. Conversely, MDA concentrations presented increased levels under 0 Pb with RBC addition rates as compared to Pb-polluted soil. It was notably observed that MDA content exhibited a decrease for Pb polluted soil without RBC amendments. From a physiological perspective, the total biomass weight was discerned to be inferior at a RBC450 level in comparison to a RBC700 concentration, whereas a decrement was evident at the 60-day relative to CN parameters (Figure 4). In the context of foliar count, *W. trilobata* exhibited an invariant response, maintaining comparable quantitative measures at both RBC450 and RBC700. The net photosynthetic rate of *W. trilobata* experienced significant fluctuations, with a pronounced reduction in activity over a 60-day duration within lead-compromised soils under both RBC450 and RBC700 addition rates.

### 2.6. Expression of Genes Encoding Antioxidant Enzymes Under Pb Stress and Biochar Amendment

**Figure 5:**
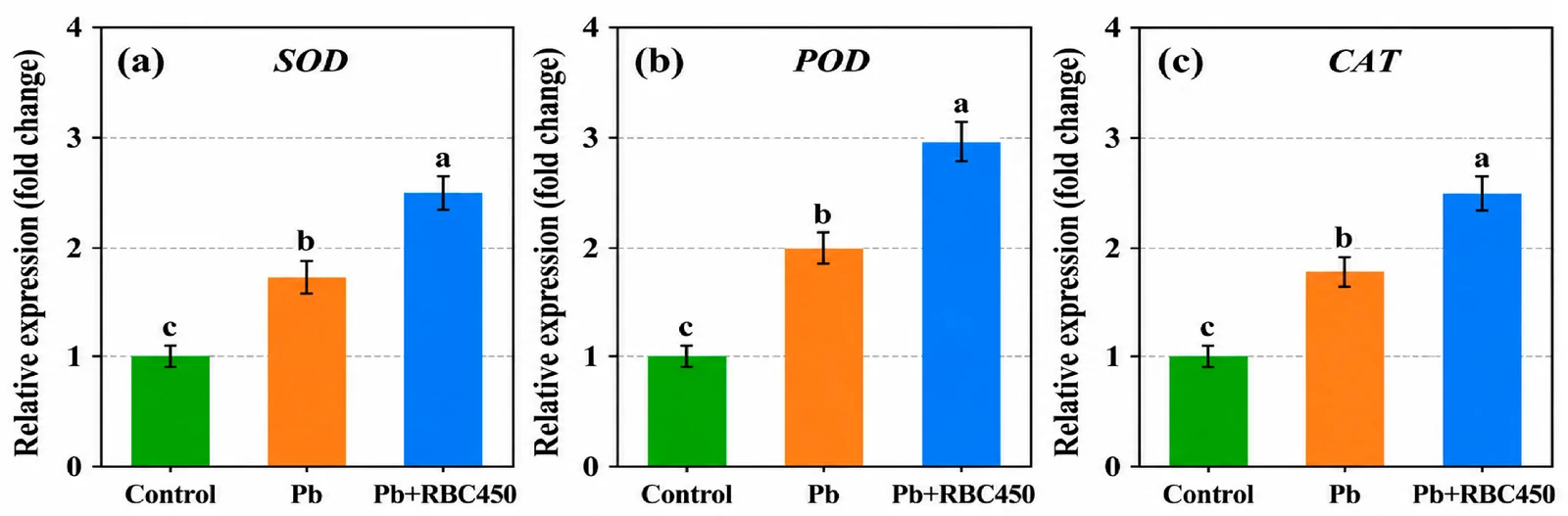
Relative transcript abundance of genes encoding (**a**) superoxide dismutase (SOD), (**b**) peroxidase (POD), and (**c**) catalase (CAT) in Wedelia trilobata under control (CN), Pb-contaminated (150 mg kg^−1^ Pb), and Pb + RBC450-5 (150 mg kg^−1^ Pb with 5% rice straw biochar produced at 450 °C) treatments. Transcript abundance was normalized using GAPDH-1 and GAPDH-2 as reference genes, with CN serving as the calibrator (relative expression = 1). Bars represent mean ± SE (n = 3 independent biological replicates). Different lowercase letters indicate statistically significant differences among treatments (*p* < 0.05).

Quantitative real-time PCR (qRT-PCR) analysis revealed significant changes in the relative transcript abundance of genes encoding superoxide dismutase (SOD), peroxidase (POD), and catalase (CAT) under Pb stress and biochar amendment (Figure 5). Compared with the untreated control, Pb exposure significantly increased the transcript abundance of the selected SOD-, POD-, and CAT-related genes, indicating transcriptional activation of antioxidant defense pathways. Among the examined genes, the POD-related gene exhibited the greatest relative induction under Pb stress, followed by the CAT- and SOD-related genes. Application of rice straw biochar (RBC450) further enhanced the relative transcript abundance of all three antioxidant-related genes compared with Pb treatment alone. The highest transcript levels were observed in the Pb + RBC450 treatment, suggesting that biochar amendment modulated antioxidant-related transcriptional responses under Pb stress. Overall, these findings demonstrate that Pb exposure and RBC450 amendment influence the transcriptional regulation of antioxidant defense-associated genes in *W. trilobata*. However, increased transcript abundance does not necessarily indicate a proportional increase in antioxidant enzyme activity.

## 3. Discussion

### 3.1. Effects of Biochar on Soil Properties and Pb Immobilization

The present study demonstrated that rice straw biochar modified soil physicochemical properties and reduced Pb availability, with responses varying according to pyrolysis temperature and amendment rate. Both RBC450 and RBC700 increased soil pH, electrical conductivity (EC), organic carbon (OC), and selected nutrient concentrations. After 60 days, soil pH increased from approximately 6.7 in the unamended control to 7.68 and 7.76 under 5% RBC450 and RBC700, respectively. These findings are consistent with previous studies demonstrating that biochar can modify soil chemical conditions and influence heavy metal mobility [21,22,23]. The increase in pH may have contributed to reduced Pb availability by promoting sorption onto soil constituents and favoring the formation of less soluble Pb compounds. However, because Pb speciation was not directly investigated, these processes remain plausible rather than experimentally confirmed immobilization mechanisms. Pyrolysis temperature influenced the characteristics and performance of the two biochars. RBC700 generally produced greater increases in soil pH and stronger reductions in extractable Pb than RBC450. Dissolved organic carbon (DOC) decreased from 8.32 mg C L^−1^ in RBC450 to 2.14 mg C L^−1^ in RBC700, indicating differences in water-extractable carbon associated with pyrolysis temperature. These differences may influence metal–organic matter interactions, although their specific contributions to Pb retention were not directly established. Biochar application also increased soil OC, nitrate nitrogen (NO_3_^−^-N), and available phosphorus (AP), whereas ammonium nitrogen (NH_4_^+^-N) generally decreased with increasing amendment rates. These changes suggest modifications in soil carbon and nutrient dynamics, potentially reflecting differences in nutrient retention, microbial transformations, and plant uptake [16,24,25]. However, the underlying nitrogen transformation processes were not independently measured. A major finding was the progressive reduction in soil-extractable Pb with increasing biochar application rates. Pb concentrations declined to 50.5, 46.8, and 35.2 mg kg^−1^ under 1%, 2.5%, and 5% RBC450, respectively, compared with 49.7, 40.6, and 26.4 mg kg^−1^ under the corresponding RBC700 treatments. At the 5% application rate, DTPA-extractable Pb decreased to 35.2 mg kg^−1^ under RBC450 and 26.4 mg kg^−1^ under RBC700, representing a 25.0% lower concentration with RBC700 than RBC450. This finding indicates greater effectiveness of RBC700 in reducing the extractable Pb fraction under the experimental conditions. Previous studies have identified surface sorption, complexation, ion exchange, and precipitation as potential mechanisms of biochar-mediated Pb retention [26,27,28,29]. Nevertheless, these processes were not independently quantified in the present study. Therefore, the observed reduction in DTPA-extractable Pb is best interpreted as evidence of decreased operationally defined Pb extractability following biochar amendment, rather than direct confirmation of a specific immobilization mechanism. Furthermore, immobilization does not represent complete Pb removal, and its long-term stability remains uncertain.

### 3.2. Pb Accumulation, Plant Physiological Responses, and Antioxidant Defense

The reduction in soil-extractable Pb was accompanied by decreased Pb accumulation in *W. trilobata*, indicating that biochar amendment reduced plant exposure to the contaminant. Pb concentrations remained higher in roots than in shoots, suggesting preferential retention in belowground tissues. Both biochars reduced tissue Pb concentrations relative to the unamended Pb treatment, with RBC700 generally producing greater reductions in root Pb accumulation. These observations are consistent with previous studies reporting that biochar can restrict heavy metal uptake by reducing the available metal fraction in soil [30,31,32]. However, root surface adsorption, intracellular sequestration, and restricted root-to-shoot transport were not independently examined; therefore, the observed Pb distribution cannot be attributed to a specific physiological mechanism. Plant growth and photosynthetic responses varied with biochar type, application rate, and Pb contamination (Figure 3). Under Pb stress, RBC700 generally supported higher biomass accumulation than RBC450, whereas changes in leaf number were treatment-dependent. Net photosynthetic rate remained lower under Pb-contaminated conditions than under the corresponding Pb-free treatments, indicating that reduced soil-extractable Pb did not completely restore photosynthetic performance. These findings indicate that decreased Pb availability did not uniformly restore plant growth and physiological activity. Pb toxicity can interfere with photosynthesis, nutrient homeostasis, and cellular metabolism, while changes in soil chemistry following biochar application may independently influence plant performance [33,34,35]. Consequently, improved Pb immobilization should not automatically be interpreted as complete alleviation of physiological stress. The biochemical responses revealed complex interactions between Pb exposure and biochar amendment (Figure 3 and Figure 4). POD and CAT activities generally increased under several biochar treatments, whereas MDA and ROS levels exhibited treatment-dependent variation. Notably, several Pb-contaminated treatments showed lower MDA and ROS levels than their corresponding Pb-free treatments, indicating that oxidative responses were not uniformly proportional to Pb exposure. Previous studies on *W. trilobata* have similarly demonstrated that antioxidant enzyme activities and oxidative stress indicators vary in response to environmental stress and physiological acclimation [36,37]. These differences may reflect changes in antioxidant regulation and physiological acclimation, although the underlying mechanisms were not directly investigated. Furthermore, increased antioxidant enzyme activities may represent an adaptive response to oxidative stress rather than complete stress alleviation. Therefore, the combined interpretation of ROS, MDA, POD, and CAT provides a more balanced assessment of biochar-associated changes in plant oxidative status.

### 3.3. Antioxidant Gene Expression and Integrated Soil–Plant Responses

**Figure 6:**
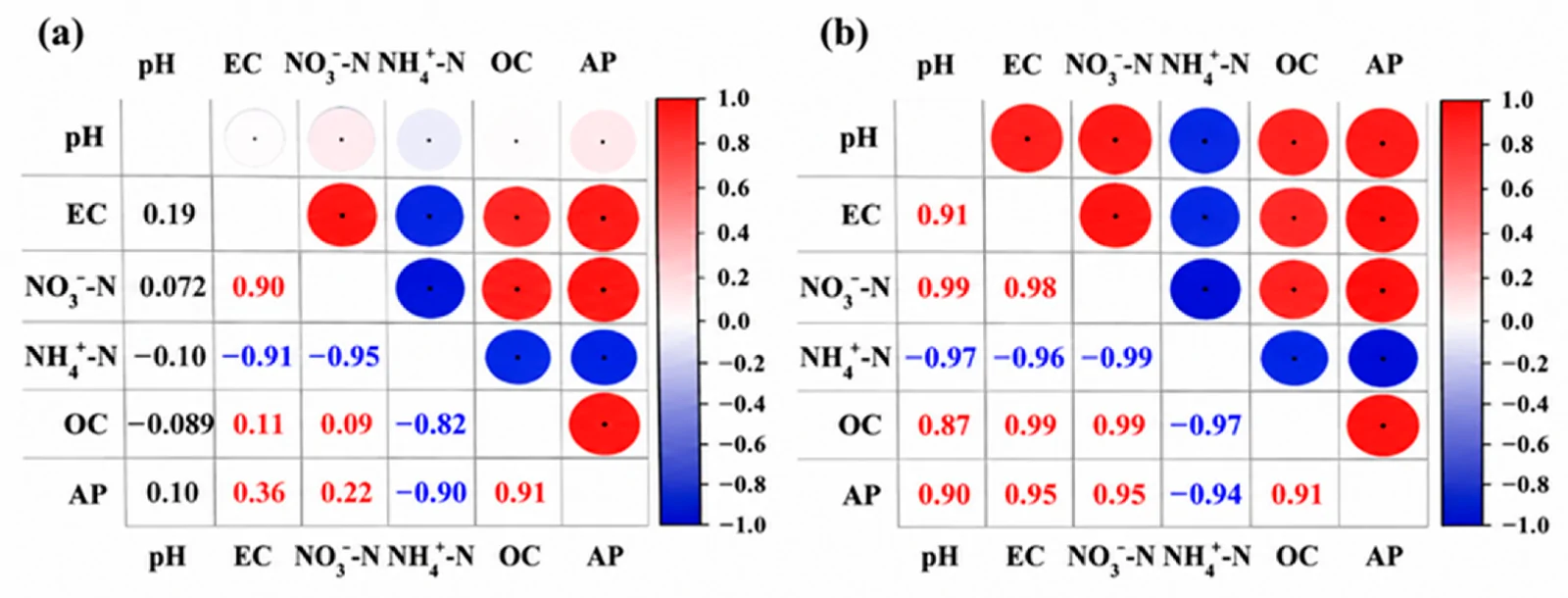
Pearson correlation matrices illustrating relationships among soil physicochemical properties under (**a**) RBC450 and (**b**) RBC700 treatments. The analyzed variables include soil pH, electrical conductivity (EC), nitrate nitrogen (NO_3_^−^-N), ammonium nitrogen (NH_4_^+^-N), organic carbon (OC), and available phosphorus (AP). Red and blue circles represent positive and negative Pearson correlation coefficients, respectively, while circle size indicates the absolute strength of the correlation. Numerical values represent Pearson correlation coefficients (r).

**Figure 7:**
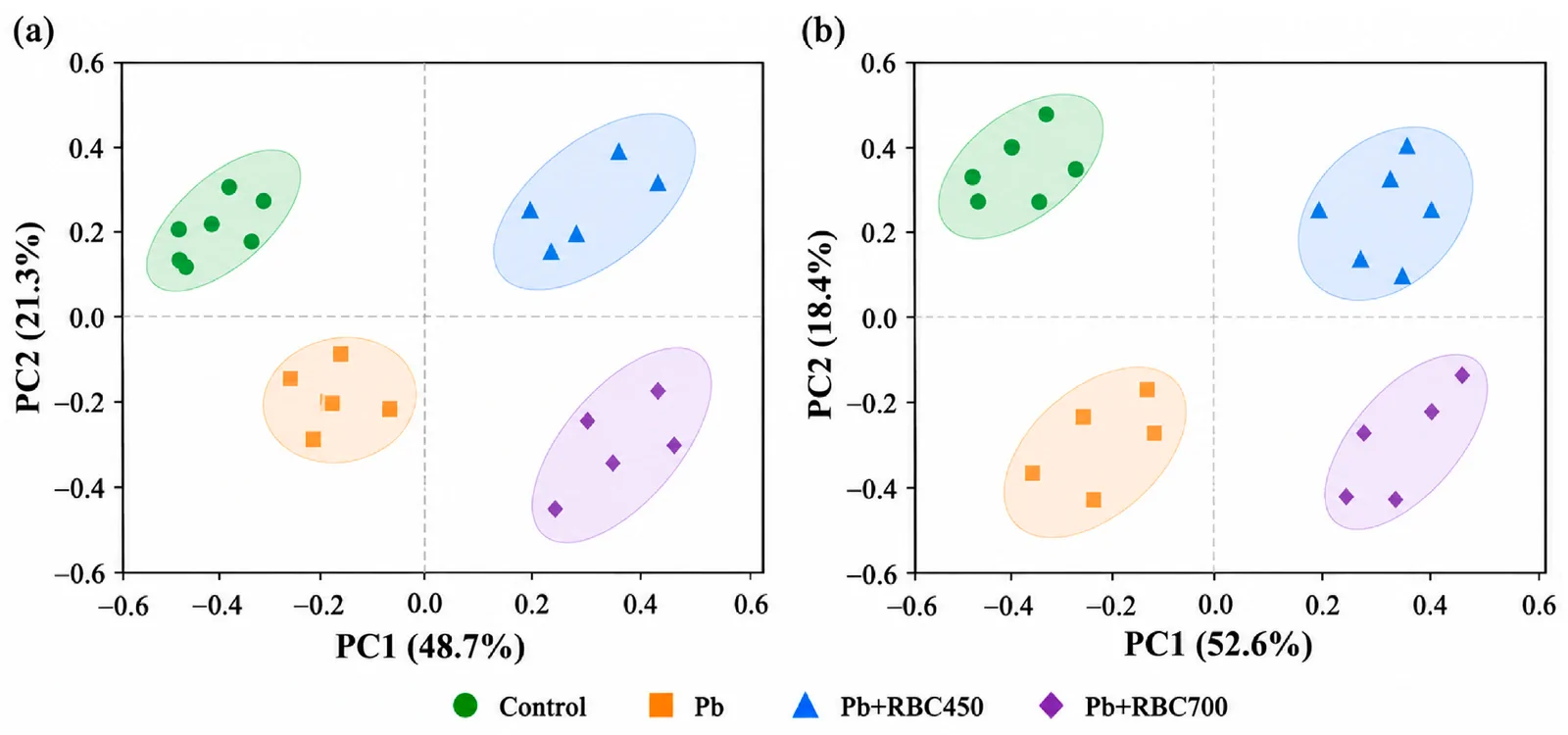
Principal component analysis (PCA) showing the distribution of experimental observations according to soil physicochemical properties under Pb contamination and rice straw biochar amendment. Panels (**a**,**b**) present PCA score plots, with the first two principal components explaining 70.0% and 71.0% of the total variance, respectively. CN represents the Pb-free control, Pb represents soil contaminated with 150 mg kg^−1^ Pb without biochar, and Pb-RBC450 and Pb-RBC700 represent Pb-contaminated groups amended with rice straw biochar produced at 450 °C and 700 °C, respectively. Shaded ellipses illustrate within-group dispersion and should not be interpreted as confidence intervals unless statistically verified.

Transcriptional analysis revealed changes in antioxidant-related gene expression under Pb stress and RBC450 amendment. The relative transcript abundance of genes encoding superoxide dismutase (SOD), peroxidase (POD), and catalase (CAT) was evaluated in three treatments: the Pb-free control (CN), the Pb-contaminated control (Pb), and Pb-contaminated soil amended with 5% RBC450 (Pb-RBC450-5). Compared with the uncontaminated control, Pb exposure increased the relative expression of the selected SOD-, POD-, and CAT-related genes, while 5% RBC450 amendment further enhanced their transcript abundance under Pb stress (Figure 5). These findings suggest that Pb exposure and biochar amendment modulated antioxidant-related transcriptional responses in *W. trilobata*. These genes encode enzymes involved in superoxide conversion and hydrogen peroxide detoxification [38,39,40]. However, transcript abundance does not necessarily correspond directly to enzyme activity because post-transcriptional regulation may influence functional antioxidant responses. Importantly, molecular analysis was conducted only for RBC450, whereas RBC700 demonstrated greater effectiveness in reducing soil-extractable Pb. Therefore, the present results cannot establish whether RBC700 would induce stronger or weaker antioxidant gene expression than RBC450. The molecular findings specifically demonstrate transcriptional changes associated with the selected 5% RBC450 treatment rather than a general pyrolysis-temperature-dependent molecular mechanism. Pearson correlation analysis identified positive and negative associations among soil pH, electrical conductivity (EC), organic carbon (OC), nitrate nitrogen (NO_3_^−^-N), ammonium nitrogen (NH_4_^+^-N), and available phosphorus (AP) across the experimental observations (Figure 6). These relationships suggest that variation in soil chemical properties was interconnected rather than occurring independently. Principal component analysis (PCA) further revealed differences in the multivariate distribution of control, Pb-contaminated, and biochar-amended treatments (Figure 7). The first two principal components explained 70.0% and 71.0% of the total variance in Figure 7a,b, respectively. The separation between RBC450- and RBC700-amended treatments suggests that pyrolysis temperature was associated with distinct patterns of soil chemical responses. Collectively, these analyses indicate that biochar amendment was associated with coordinated changes in soil physicochemical properties, with response patterns differing between RBC450 and RBC700. However, these statistical associations do not establish causality or identify the specific mechanisms responsible for the observed changes. Overall, RBC700 demonstrated greater effectiveness in reducing soil DTPA-extractable Pb, whereas antioxidant-related gene expression was investigated only under the 5% RBC450 treatment. Therefore, the molecular findings cannot establish whether RBC700 would induce stronger or weaker antioxidant responses than RBC450. Moreover, potential mechanisms involving surface complexation, ion exchange, and Pb precipitation remain hypothetical because the relevant biochar characteristics and Pb species were not directly determined. These findings highlight the need for comparative molecular analyses and comprehensive biochar characterization before drawing broader conclusions about the relative performance of the two materials.

## 4. Materials and Methods

### 4.1. Biochar Preparation and Characterization

Rice straw was used as the feedstock for biochar production. The collected biomass was cleaned, dried, and subsequently subjected to pyrolysis in a muffle furnace (SX2-12-10, Ningbo Hinotek Technology Co., Ningbo, China) under oxygen-limited conditions at two temperatures, 450 °C and 700 °C, for 6 h. Following pyrolysis, the resulting biochars were crushed and passed through a 2 mm sieve. The materials were designated RBC450 and RBC700 according to their respective pyrolysis temperatures. A portion of each biochar was subsequently processed using a high-energy planetary ball mill (QM-3SP2, T-Bota Scitech, Nanjing, China) at a reported frequency of 40 Hz for 8 h to reduce particle size. The surface morphology and elemental composition of RBC450 and RBC700 were examined using scanning electron microscopy coupled with energy-dispersive X-ray spectroscopy (SEM–EDX; FEI Quanta 200FEG, Hillsboro, OR, USA). SEM imaging was used to characterize surface morphology, particle structure, and visible pore features, whereas EDX analysis was used to assess the elemental composition of the biochar surfaces. Carbon (C), hydrogen (H), and nitrogen (N) contents were determined using an elemental analyzer (MicroCube, Elementar, Germany), while oxygen (O) content was estimated by mass balance. RBC450 and RBC700 were incorporated into the experimental soils at application rates of 1%, 2.5%, and 5% (*w*/*w*) to evaluate their effects on Pb immobilization, soil physicochemical properties, and plant responses. Additional physicochemical characteristics of RBC450 and RBC700, including the reported surface area, pH, electrical conductivity, ash content, and dissolved organic carbon (DOC), are summarized in Supplementary Table S1. Although surface-area values are provided, the original documentation does not specify the analytical instrument or measurement conditions used for their determination. Therefore, these values are reported descriptively and are not identified as independently verified BET-specific surface-area measurements. The available SEM–EDX analyses were interpreted only in terms of observable morphology and elemental composition, without inferring specific surface functional groups or Pb-binding mechanisms.

### 4.2. Pot Experiment

The pot experiment was conducted in the greenhouse facility of Jiangsu University, Zhenjiang, Jiangsu Province, China, to evaluate the effects of rice straw biochar produced at two pyrolysis temperatures, 450 °C (RBC450) and 700 °C (RBC700), on soil physicochemical properties, Pb immobilization, and the physiological, biochemical, and molecular responses of *Wedelia trilobata* (L.) Hitchc. under Pb stress. Plant materials were collected from multiple locations in Jiangsu Province during March–April 2024 and identified based on morphological and taxonomic characteristics by the Seed Bank of Jiangsu University, China. The collected plants were propagated under controlled conditions, and healthy, uniform, one-month-old vegetative cuttings (6–8 cm long) were selected for transplantation. No voucher specimen was deposited. The experiment comprised 14 treatments, including two controls without biochar: CN (0 mg Pb kg^−1^ soil) and Pb (150 mg Pb kg^−1^ soil). Lead contamination was established using lead (II) nitrate [Pb (NO_3_)_2_], with the nominal Pb addition rate expressed on an elemental Pb basis. For each biochar type, three application rates (1%, 2.5%, and 5%, *w*/*w*) were evaluated under Pb-free and Pb-contaminated conditions (150 mg Pb kg^−1^ soil). The RBC450 treatments were designated RBC450-1, RBC450-2.5, and RBC450-5 under Pb-free conditions, and Pb-RBC450-1, Pb-RBC450-2.5, and Pb-RBC450-5 under Pb contamination. Similarly, the RBC700 treatments were designated RBC700-1, RBC700-2.5, and RBC700-5 under Pb-free conditions, and Pb-RBC700-1, Pb-RBC700-2.5, and Pb-RBC700-5 under Pb contamination. Biochar application rates were calculated on a dry soil weight basis. Each treatment consisted of three independent plastic pots (5 L capacity), with three uniform *W. trilobata* cuttings transplanted into each pot, resulting in 42 pots and 126 plants. Each pot was considered an independent experimental unit (n = 3), whereas individual plants within the same pot were treated as subsamples to avoid pseudo replication. The prepared soils were maintained for 30 days before transplantation. Transplantation was performed in May 2024, and plants were maintained under greenhouse conditions at approximately 20–25 °C with a 12 h photoperiod. Distilled water was supplied as required, approximately every five days, to maintain soil moisture near 70% of field capacity. Soil samples were collected at 0, 20, 40, and 60 days to evaluate temporal changes in soil physicochemical properties. Leaf gas-exchange parameters and other nondestructive physiological measurements were recorded on intact plants before harvesting. Plants were harvested 60 days after transplantation, and the collected samples were subsequently used for biomass determination, biochemical assays, Pb analysis, and molecular investigations.

### 4.3. Soil Analysis

Soil physicochemical properties were analyzed to evaluate the effects of rice straw biochar amendments on soil chemical characteristics under Pb contamination. Soil pH was measured in a soil-to-deionized-water suspension (1:2.5, *w*/*v*) using a pH electrode (PE-10, Sartorius, Göttingen, Germany). Soil electrical conductivity (EC) was determined in a soil-to-water suspension prepared at a ratio of 1:5 (*w*/*v*). Soil inorganic nitrogen, including ammonium nitrogen (NH_4_^+^–N) and nitrate nitrogen (NO_3_^−^–N), was extracted using 2 M KCl and quantified by flow injection analysis (TRAACA-2000, Germany; model designation to be verified). Soil organic carbon (OC) was determined by dichromate wet oxidation using potassium dichromate (K_2_Cr_2_O_7_) and concentrated sulfuric acid (H_2_SO_4_), consistent with the principle of the Walkley–Black method. Available phosphorus (AP) was extracted using sodium bicarbonate (NaHCO_3_, pH 8.5) and subsequently quantified by colorimetric analysis. Soil OC was expressed in g kg^−1^, whereas NH_4_^+^–N, NO_3_^−^–N, and AP were expressed in mg kg^−1^ on a dry-soil basis.

### 4.4. Determination of Physiological and Antioxidant Parameters in W. trilobata

Plant growth, photosynthetic performance, and antioxidant defense responses were evaluated to investigate the effects of rice straw biochar amendments on *W. trilobata* under Pb stress. Leaf number was determined by counting the fully developed leaves on each experimental plant at the final sampling stage. Leaf area was evaluated using measurements obtained from fully expanded leaves, and the resulting values were used to compare leaf growth responses among treatments. Plant-level observations were averaged within each experimental pot, which served as the independent biological replicate (n = 3). Before harvesting, leaf gas-exchange measurements were performed on fully expanded leaves from the upper canopy of intact, living plants using an LI-6400 portable photosynthesis system (LI-COR Biosciences, Lincoln, NE, USA). Measurements were conducted between 09:00 and 11:30 h under controlled leaf-chamber conditions. During gas-exchange measurements, photosynthetically active radiation (PAR) was maintained at 800 μmol m^−2^ s^−1^ using the controlled light source of the LI-6400 system. Leaf-chamber temperature was regulated at 26–28 °C through the instrument’s temperature-control system. Reference CO_2_ concentration was monitored within the reported range of approximately 400–500 μmol mol^−1^ using the instrument’s CO_2_ regulation system, while airflow was maintained at 1000 mL min^−1^. Net photosynthetic rate (Pn, μmol CO_2_ m^−2^ s^−1^), intercellular CO_2_ concentration (Ci, μmol mol^−1^), and transpiration rate (E, mmol H_2_O m^−2^ s^−1^) were obtained from the gas-exchange measurements. Instantaneous water-use efficiency (WUE, μmol CO_2_ mmol^−1^ H_2_O) was calculated as the ratio of Pn to E. Relative leaf chlorophyll content was measured separately using FK-YL02 portable chlorophyll meter (Shandong Fangke Instrument Co., Ltd., Weifang, Shandong, China), and the results were expressed as SPAD values. The chlorophyll analyzer was not used to determine gas-exchange parameters. Following the physiological measurements, plants were harvested at the end of the 60-day experimental period. Samples intended for biomass determination were oven-dried at 72 °C for 48 h and weighed using a precision analytical balance. Fresh leaf tissues were collected separately before drying for biochemical analyses. Peroxidase (POD) activity was assessed using the guaiacol oxidation method, based on the spectrophotometric detection of tetraguaiacol formation, with 470 nm as the reference analytical wavelength. Catalase (CAT) activity was evaluated using the ultraviolet absorption method, based on the decomposition of hydrogen peroxide (H_2_O_2_), for which absorbance is conventionally monitored at 240 nm. Malondialdehyde (MDA) content, an indicator of membrane lipid peroxidation, was determined using the thiobarbituric acid (TBA) method, for which absorbance is commonly recorded at 532 nm with background correction at 600 nm. These assay principles are consistent with methodologies previously reported for *W. trilobata* [36,37,38].

### 4.5. Determination of Pb in Soil and Plant Tissues

Total lead (Pb) concentrations in soil and plant tissues were determined using microwave-assisted acid digestion followed by atomic absorption spectrometry (AAS). For sample mineralization, approximately 0.1 g of air-dried soil or 0.2 g of dried plant tissue was transferred into microwave digestion vessels and treated with a mixture of 6 mL nitric acid (HNO_3_) and 2 mL hydrofluoric acid (HF). Digestion was performed using a MARS 6 microwave digestion system (CEM Corporation, Matthews, NC, USA). Separate digestion temperature programs were applied to soil and plant samples. Soil samples were heated sequentially to 130 °C for 6 min, 150 °C for 8 min, and 205 °C for 35 min, whereas plant samples were heated to 130 °C for 8 min, 150 °C for 4 min, and 180 °C for 10 min. Following digestion, the resulting solutions were evaporated to near dryness at 150 °C and subsequently diluted with deionized water before Pb determination using AAS. Total Pb concentrations were expressed as mg kg^−1^ dry weight. The DTPA-extractable Pb fraction in soil was determined separately using a diethylenetriaminepentaacetic acid (DTPA)-based extraction procedure. Following extraction, Pb concentrations in the resulting solutions were quantified using AAS to assess changes in the operationally defined extractable Pb fraction associated with biochar application. DTPA-extractable Pb concentrations were expressed as mg kg^−1^ dry soil. The acid digestion procedure was used to quantify total Pb concentrations in plant tissues. Therefore, the measured Pb concentrations represent total tissue Pb rather than specific bioavailable or chemically fractionated Pb forms. Individual Pb species and their subcellular distributions were not determined in this study.

### 4.6. Gene Expression Analysis (qRT-PCR)

The relative transcript abundance of antioxidant-related genes in *W. trilobata* was evaluated using quantitative reverse transcription polymerase chain reaction (qRT-PCR). Fresh leaf samples were collected after 60 days of exposure from three experimental groups: (i) untreated control (CN; 0 mg kg^−1^ Pb without biochar), (ii) Pb-contaminated control (Pb; 150 mg kg^−1^ Pb without biochar), and (iii) Pb-contaminated soil amended with 5% (*w*/*w*) rice straw biochar produced at 450 °C (Pb + RBC450-5). Three independent biological replicates were included per treatment, with each replicate representing an independent experimental pot. Total RNA was extracted from fresh leaf tissues using a commercial plant RNA extraction kit according to the manufacturer’s instructions. RNA concentration and integrity were assessed using spectrophotometry and agarose gel electrophoresis, respectively. Residual genomic DNA was removed by DNase treatment, and first-strand complementary DNA (cDNA) was synthesized using a reverse transcription kit according to the manufacturer’s protocol. Quantitative real-time PCR was performed using SYBR Green-based detection chemistry with gene-specific primers targeting transcripts encoding superoxide dismutase (SOD), peroxidase (POD), and catalase (CAT). Two reference genes, glyceraldehyde-3-phosphate dehydrogenase 1 (GAPDH-1) and glyceraldehyde-3-phosphate dehydrogenase 2 (GAPDH-2), were used for normalization. The corresponding primer sequences and characteristics are presented in Table 3. Each biological replicate was analyzed in three technical PCR replicates. The amplification protocol consisted of an initial denaturation step followed by 40 amplification cycles. Primer specificity was assessed by melting-curve analysis, which revealed single distinct peaks for the analyzed primer pairs. Relative transcript abundance was calculated using the 2^−^ΔΔCt method, with the untreated control serving as the calibrator. Differences in relative transcript abundance among treatments were evaluated using one-way analysis of variance (ANOVA), followed by Tukey’s multiple-comparison test. Statistical significance was established at *p* < 0.05.

### 4.7. Statistical Analysis

Statistical analyses were performed using OriginPro 2021b (OriginLab Corporation, Northampton, MA, USA). The experiment comprised 14 treatment combinations, each with three independent biological replicates, resulting in 42 experimental pots. Each pot was considered an independent experimental unit (n = 3), whereas individual plants within the same pot were treated as subsamples. Plant-level measurements were averaged within each pot before statistical analysis to avoid pseudo replication. For soil physicochemical properties measured at 0, 20, 40, and 60 days, treatment comparisons were conducted separately for each sampling day using the available treatment means, standard deviations, and three independent pots per treatment. Because the original pot-level longitudinal dataset was unavailable, repeated-measures ANOVA could not be performed, and treatment-by-time interactions were not evaluated. Accordingly, the day-specific comparisons were interpreted as exploratory analyses rather than evidence of longitudinal treatment effects. These comparisons employed summary-statistics-based one-way ANOVA followed by Tukey’s honestly significant difference (HSD) test. For plant growth, photosynthetic characteristics, antioxidant enzyme activities, oxidative stress indicators, and Pb accumulation measured at the final harvest, differences among the 14 treatment combinations were evaluated using one-way ANOVA. Relative transcript abundance of antioxidant-related genes was evaluated separately using one-way ANOVA across three experimental groups: untreated control (CN), Pb-contaminated control (Pb), and Pb-contaminated soil amended with 5% RBC450 (Pb + RBC450-5). Where significant treatment effects were identified, Tukey’s honestly significant difference (HSD) test was used for multiple comparisons. The normality of residuals and homogeneity of variance were assessed to evaluate the assumptions underlying parametric analyses. Principal component analysis (PCA) was employed to investigate multivariate patterns in soil physicochemical properties across experimental treatments. Pearson’s correlation analysis was performed to assess linear relationships among soil pH, electrical conductivity (EC), organic carbon (OC), nitrate nitrogen (NO_3_^−^–N), ammonium nitrogen (NH_4_^+^–N), and available phosphorus (AP). Correlation matrices were visualized using the Correlation Plot App in OriginPro 2021b. Statistical significance was established at *p* < 0.05, and experimental results were expressed as mean ± standard deviation (SD) or standard error (SE), as specified in the corresponding tables and figure legends.

## 5. Conclusions

This study demonstrates the potential of rice straw-derived biochar to mitigate Pb toxicity by improving soil physicochemical properties, restricting Pb accumulation, and enhancing physiological and molecular defense responses in *W. trilobata*. Biochar amendments modified soil pH, electrical conductivity, organic carbon, and nutrient availability, creating conditions that may favor Pb immobilization and improved plant performance. Notably, RBC700 exhibited greater effectiveness than RBC450 in reducing Pb mobility and accumulation, showing the importance of pyrolysis temperature in determining remediation efficiency. Biochar application also alleviated Pb-induced oxidative stress by enhancing antioxidant enzyme activities, particularly catalase (CAT) and peroxidase (POD) while reducing malondialdehyde (MDA) accumulation and associated lipid peroxidation. Furthermore, qRT-PCR analysis revealed differential regulation of antioxidant-related genes (*SOD*, *POD*, and *CAT*), suggesting that biochar-mediated stress alleviation involves coordinated biochemical and molecular responses. Collectively, these findings indicate that rice straw biochar can simultaneously influence Pb behavior in soil and improve plant tolerance to metal-induced oxidative stress. The superior performance of RBC700 further suggests that pyrolysis conditions are critical for optimizing biochar-based remediation strategies. Future studies should evaluate the long-term stability of immobilized Pb, biochar aging, and field-scale performance to establish the practical applicability of this approach for sustainable remediation of Pb-contaminated soils.

## Figures and Tables

**Table 1:** Effects of rice straw biochar on soil physicochemical properties (pH, EC, and OC) under Pb contamination.

Parameter	Day	CN Pb 0	CN Pb 150	RBC450 1% Pb 0	RBC450 1% Pb 150	RBC450 2.5% Pb 0	RBC450 2.5% Pb 150	RBC450 5% Pb 0	RBC450 5% Pb 150	RBC700 1% Pb 0	RBC700 1% Pb 150	RBC700 2.5% Pb 0	RBC700 2.5% Pb 150	RBC700 5% Pb 0	RBC700 5% Pb 150
pH	0	6.72 ± 0.31 a	6.77 ± 0.72 a	6.78 ± 0.52 a	6.74 ± 0.31 a	6.73 ± 0.37 a	6.77 ± 0.54 a	6.82 ± 0.89 a	6.78 ± 0.74 a	6.74 ± 0.47 a	6.73 ± 0.53 a	6.77 ± 0.47 a	6.75 ± 0.62 a	6.81 ± 0.51 a	6.77 ± 0.73 a
pH	20	6.74 ± 0.28 a	6.72 ± 0.36 a	6.94 ± 0.42 a	7.12 ± 0.53 a	6.99 ± 0.42 a	7.05 ± 0.44 a	7.24 ± 0.25 a	7.32 ± 0.54 a	6.92 ± 0.25 a	6.93 ± 0.62 a	6.99 ± 0.25 a	6.99 ± 0.25 a	7.31 ± 0.62 a	7.13 ± 0.34 a
pH	40	6.7 ± 0.45 a	6.7 ± 0.63 a	7.05 ± 0.33 a	7.19 ± 0.16 a	7.28 ± 0.63 a	7.27 ± 0.14 a	7.52 ± 0.32 a	7.54 ± 0.14 a	7.14 ± 0.33 a	7.03 ± 0.16 a	7.35 ± 0.43 a	7.32 ± 0.19 a	7.57 ± 0.43 a	7.53 ± 0.65 a
pH	60	6.73 ± 0.54 ab	6.68 ± 0.36 b	7.14 ± 0.38 ab	7.21 ± 0.26 ab	7.37 ± 0.22 ab	7.39 ± 0.29 ab	7.71 ± 0.48 ab	7.68 ± 0.62 ab	7.22 ± 0.19 ab	7.16 ± 0.31 ab	7.54 ± 0.22 ab	7.49 ± 0.49 ab	7.85 ± 0.22 a	7.76 ± 0.43 ab
EC	0	0.22 ± 0.03 a	0.21 ± 0.03 a	0.21 ± 0.04 a	0.21 ± 0.02 a	0.2 ± 0.04 a	0.19 ± 0.02 a	0.22 ± 0.04 a	0.23 ± 0.04 a	0.21 ± 0.01 a	0.22 ± 0.01 a	0.22 ± 0.01 a	0.21 ± 0.02 a	0.25 ± 0.02 a	0.23 ± 0.04 a
EC	20	0.21 ± 0.04 e	0.21 ± 0.04 e	0.24 ± 0.02 de	0.32 ± 0.04 cd	0.32 ± 0.01 cd	0.34 ± 0.03 abc	0.42 ± 0.03 ab	0.37 ± 0.01 abc	0.33 ± 0.03 bcd	0.32 ± 0.04 cd	0.34 ± 0.04 abc	0.29 ± 0.03 cde	0.43 ± 0.04 a	0.32 ± 0.03 cd
EC	40	0.21 ± 0.02 e	0.19 ± 0.03 e	0.39 ± 0.06 cd	0.39 ± 0.06 cd	0.48 ± 0.03 abc	0.48 ± 0.06 abc	0.58 ± 0.01 a	0.53 ± 0.04 ab	0.48 ± 0.01 abc	0.46 ± 0.02 bcd	0.47 ± 0.05 bcd	0.37 ± 0.01 d	0.58 ± 0.01 a	0.47 ± 0.02 bcd
EC	60	0.23 ± 0.01 f	0.18 ± 0.04 f	0.54 ± 0.01 d	0.43 ± 0.01 e	0.58 ± 0.05 cd	0.59 ± 0.01 bcd	0.68 ± 0.02 a	0.67 ± 0.03 a	0.52 ± 0.02 d	0.57 ± 0.02 cd	0.53 ± 0.00 d	0.58 ± 0.02 cd	0.66 ± 0.03 ab	0.64 ± 0.01 abc
OC	0	4.52 ± 1.13 a	4.58 ± 0.82 a	4.63 ± 0.93 a	4.95 ± 0.52 a	5.13 ± 0.35 a	4.99 ± 0.41 a	4.98 ± 1.12 a	5.23 ± 0.52 a	4.89 ± 0.74 a	4.82 ± 1.02 a	5.01 ± 1.03 a	4.95 ± 0.72 a	4.67 ± 0.63 a	5.06 ± 0.89 a
OC	20	4.44 ± 0.89 d	4.59 ± 0.31 cd	6.94 ± 1.05 bcd	7.12 ± 1.31 bcd	7.73 ± 0.95 bcd	8.32 ± 1.02 bc	8.34 ± 1.41 bc	16.3 ± 1.21 a	6.92 ± 1.54 bcd	8.93 ± 0.98 b	14.6 ± 2.13 a	8.24 ± 1.02 bc	17.2 ± 1.35 a	9.44 ± 1.52 b
OC	40	4.53 ± 0.98 h	4.6 ± 0.22 h	8.34 ± 1.32 fgh	7.19 ± 3.16 gh	11.6 ± 1.41 defg	13.6 ± 2.05 def	17.3 ± 0.24 bcd	21.5 ± 3.05 ab	10.4 ± 1.12 efgh	10 ± 1.62 efgh	20.2 ± 2.63 abc	15.3 ± 2.15 cde	24.6 ± 1.88 a	22.5 ± 2.88 ab
OC	60	4.56 ± 1.02 d	4.61 ± 0.45 d	12.5 ± 2.41 cd	13.5 ± 2.04 c	18.5 ± 2.63 bc	18.4 ± 3.31 bc	26.3 ± 4.23 ab	27.5 ± 2.93 a	15.2 ± 2.62 c	16.2 ± 3.15 c	28.3 ± 3.15 a	27.3 ± 3.52 a	32.6 ± 3.04 a	30.5 ± 2.14 a

Note. Values are mean ± standard deviation (SD; n = 3 independent pots per treatment). Pb 0 and Pb 150 denote 0 and 150 mg kg^−1^ added Pb, respectively. Different lowercase letters within the same parameter and day indicate statistically significant differences by Tukey’s HSD test (adjusted *p* < 0.05); means sharing at least one letter are not significantly different. Letters were reconstructed from published summary statistics using separate one-way ANOVAs for each parameter and sampling day, not a longitudinal repeated-measures analysis. These comparisons are provisional and rely on the reported rounded SD values and independent-group assumptions.

**Table 2:** Effects of rice straw biochar on soil nutrient properties (NO_3_^−^-N, NH_4_^+^-N, and AP) under Pb contamination.

Parameter	Day	CN Pb 0	CN Pb 150	RBC450 1% Pb 0	RBC450 1% Pb 150	RBC450 2.5% Pb 0	RBC450 2.5% Pb 150	RBC450 5% Pb 0	RBC450 5% Pb 150	RBC700 1% Pb 0	RBC700 1% Pb 150	RBC700 2.5% Pb 0	RBC700 2.5% Pb 150	RBC700 5% Pb 0	RBC700 5% Pb 150
NO_3_^−^-N	0	23.4 ± 2.14 ^a^	23.5 ± 2.32 ^a^	25.6 ± 3.35 ^a^	25.8 ± 3.31 ^a^	27.4 ± 2.34 ^a^	26.3 ± 3.42 ^a^	26.4 ± 2.54 ^a^	26.3 ± 2.57 ^a^	26.5 ± 3.64 ^a^	27.4 ± 2.34 ^a^	27.3 ± 2.47 ^a^	27.6 ± 3.53 ^a^	26.3 ± 2.51 ^a^	26.7 ± 5.23 ^a^
NO_3_^−^-N	20	23.9 ± 3.04 ^e^	24.4 ± 1.98 ^e^	45.6 ± 4.36 ^bcd^	43.7 ± 4.52 ^bcd^	49.2 ± 5.24 ^bc^	44.6 ± 2.54 ^bcd^	54.9 ± 3.56 ^ab^	62.6 ± 3.63 ^a^	33.7 ± 6.24 ^de^	36.6 ± 4.63 ^d^	37.4 ± 4.25 ^cd^	42.6 ± 2.53 ^cd^	37.6 ± 4.62 ^cd^	42.5 ± 3.76 ^cd^
NO_3_^−^-N	40	24.1 ± 1.45 ^d^	24.2 ± 2.53 ^d^	52.6 ± 5.28 ^bc^	53.6 ± 5.16 ^bc^	60.3 ± 6.18 ^bc^	62.6 ± 7.46 ^b^	66.8 ± 7.85 ^ab^	77.1 ± 4.14 ^a^	52.5 ± 4.53 ^bc^	47.5 ± 3.15 ^c^	55.3 ± 4.33 ^bc^	55.6 ± 3.78 ^bc^	56.3 ± 3.36 ^bc^	57.4 ± 3.65 ^bc^
NO_3_^−^-N	60	23.9 ± 1.56 ^d^	23.9 ± 2.85 ^d^	68.9 ± 4.59 ^c^	69.5 ± 3.96 ^c^	82.5 ± 5.37 ^abc^	81.5 ± 11.7 ^abc^	89.3 ± 6.35 ^ab^	90.5 ± 9.31 ^ab^	72.6 ± 5.63 ^bc^	77.2 ± 5.24 ^abc^	80.3 ± 2.19 ^abc^	80.2 ± 8.85 ^abc^	93.6 ± 7.95 ^a^	92.4 ± 6.35 ^a^
NH_4_^+^-N	0	39.1 ± 3.23	40.2 ± 2.53	40.3 ± 2.63	40.3 ± 3.72	38.6 ± 3.73	38.8 ± 4.14	39.5 ± 2.74	39.4† 5.34	39.8 ± 2.51	39.7 ± 2.53	38.9 ± 3.14	38.6 ± 3.53	40.1 ± 3.51	38.7 ± 5.13
NH_4_^+^-N	20	40.1 ± 4.13 ^a^	39.8 ± 4.63 ^ab^	31.6 ± 5.23 ^abc^	32.7 ± 4.82 ^abc^	26.6 ± 3.11 ^c^	29.7 ± 3.62 ^abc^	27.3 ± 3.83 ^c^	27.2 ± 4.32 ^c^	29.3 ± 1.31 ^bc^	28.4 ± 2.62 ^c^	27.8 ± 2.25 ^c^	26.4 ± 2.42 ^c^	27.5 ± 1.62 ^c^	26.3 ± 3.34 ^c^
NH_4_^+^-N	40	39.7 ± 2.65 ^a^	40.2 ± 2.64 ^a^	21.6 ± 3.63 ^b^	20.5 ± 2.47 ^b^	17.3 ± 2.94 ^b^	17.3 ± 2.62 ^b^	17.7 ± 1.13 ^b^	18.3 ± 3.22 ^b^	19.3 ± 3.24 ^b^	21.6 ± 1.16 ^b^	18.6 ± 2.33 ^b^	18.4 ± 1.75 ^b^	16.8 ± 2.63 ^b^	17.2 ± 0.62 ^b^
NH_4_^+^-N	60	39.9 ± 1.74 ^a^	40.4 ± 1.99 ^a^	7.14 ± 1.21 ^b^	8.25 ± 2.16 ^b^	6.45 ± 1.63 ^b^	7.33 ± 2.51 ^b^	5.72 ± 2.61 ^b^	7.35 ± 9.84 ^b^	9.24 ± 1.13 ^b^	7.16 ± 0.98 ^b^	7.73 ± 1.23 ^b^	6.46 ± 1.13 ^b^	7.85 ± 1.22 ^b^	4.63 ± 1.03 ^b^
AP	0	28.5 ± 2.13 ^a^	29.1 ± 2.13 ^a^	28.9 ± 3.53 ^a^	29.4 ± 0.52 ^a^	30.5 ± 1.32 ^a^	29.4 ± 2.41 ^a^	30.2 ± 1.12 ^a^	29.5 ± 2.73 ^a^	28.9 ± 3.02 ^a^	30.5 ± 2.53 ^a^	30.1 ± 1.87 ^a^	29.5 ± 1.45 ^a^	29.5 ± 2.74 ^a^	30.4 ± 2.36 ^a^
AP	20	28.8 ± 1.45 ^cd^	28.5 ± 1.64 ^d^	32.5 ± 2.52 ^bcd^	32.5 ± 3.31 ^bcd^	36.6 ± 3.52 ^ab^	32.5 ± 1.02 ^bcd^	39.5 ± 2.41 ^a^	35.2 ± 1.02 ^abc^	36.9 ± 2.36 ^ab^	40.5 ± 0.99 ^a^	34.6 ± 1.09 ^abcd^	35.2 ± 2.64 ^abc^	37.4 ± 1.47 ^ab^	37.3 ± 2.88 ^ab^
AP	40	29.4 ± 2.43 ^e^	29.4 ± 2.64 ^e^	38.7 ± 1.63 ^d^	38.4 ± 2.16 ^d^	42.5 ± 2.64 ^cd^	38.4 ± 4.05 ^d^	52.5 ± 2.09 ^a^	44.6 ± 3.07 ^bcd^	40.4 ± 1.08 ^d^	41.5 ± 3.28 ^d^	50.2 ± 2.75 ^abc^	43.1 ± 2.15 ^bcd^	44.5 ± 2.98 ^bcd^	50.3 ± 1.95 ^ab^
AP	60	29.5 ± 2.54 ^c^	29.6 ± 3.05 ^c^	45.7 ± 4.25 ^b^	44.5 ± 5.01 ^b^	54.3 ± 2.95 ^ab^	44.5 ± 3.31 ^b^	60.6 ± 5.43 ^a^	59.3 ± 6.13 ^a^	43.2 ± 3.18 ^b^	52.6 ± 2.14 ^ab^	52.3 ± 6.88 ^ab^	55.2 ± 3.52 ^ab^	56.3 ± 4.37 ^ab^	63.6 ± 5.53 ^a^

Note. Values are presented as mean ± standard deviation (SD; n = 3 independent pots per treatment). Pb 0 and Pb 150 indicate the addition of 0 and 150 mg Pb kg^−1^ soil, respectively. Different lowercase superscript letters within the same parameter and sampling day indicate statistically significant differences among treatments according to one-way ANOVA followed by Tukey’s HSD test (adjusted *p* < 0.05). Values sharing at least one letter are not significantly different. Statistical comparisons were reconstructed from rounded summary statistics and performed separately for each sampling day; therefore, they do not account for within-pot correlations across repeated measurements. The two anomalous NH_4_^+^–N entries could not be independently verified from the original replicate-level data and were excluded from the affected significance comparison.

**Table 3:** Primer details used for qRT-PCR analysis of antioxidant genes in *W. trilobata.*

Gene	Forward Primer (5′–3′)	Reverse Primer (5′–3′)	Forward Length (bp)	Reverse Length (bp)	Forward GC (%)
SOD	TGGTTTGAAAGCGGTGG	CTGGTTTAAGCCCTGTGAT	17	19	52.9
POD	CAACACCGCAGAGAAAGACT	CTGGGAGGTAAAGAGAAC	20	18	50.0
CAT	CAAGACCTGGCCTGAG	TGTCTCTGAGTGTCCG	16	16	62.5
GAPDH-1	CTGCTTCATTCAACATC	CTCACGGTCAGATCAACA	17	18	41.2
GAPDH-2	GGCTCGACTCGGCATATTCT	CGGCTGCCTTTGGTCTATGT	20	20	55.0

## Data Availability

All data generated or analyzed during this study are included in this article.

## Supplementary Materials

The following supporting information can be downloaded at https://www.mdpi.com/article/10.3390/plants15213445/s1, Figure S1: qRT-PCR amplification curves (SOD, POD, CAT, GAPDH-1, GAPDH-2) showing sigmoidal amplification profiles, and melting curves confirming single specific peaks for all genes in *Wedelia trilobata*. Data indicate efficient amplification and primer specificity. Table S1: Physicochemical characteristics of rice straw biochar produced at 450 °C (RBC450) and 700 °C (RBC700).
