# Supplementary material for: Pyrolysis Temperature-Dependent Biochar Regulates Lead Immobilization and Antioxidant Defense in Wedelia trilobata for Contaminated Soil Remediation
Source: Plants (Basel). 2026 Nov 5;15(21):3445. doi: 10.3390/plants15213445 (PMC13765548; doi:10.3390/plants15213445)
Supplement: Supplementary file 1 [file plants-15-03445-s001.zip › plants-4621281-supplementary.pdf]

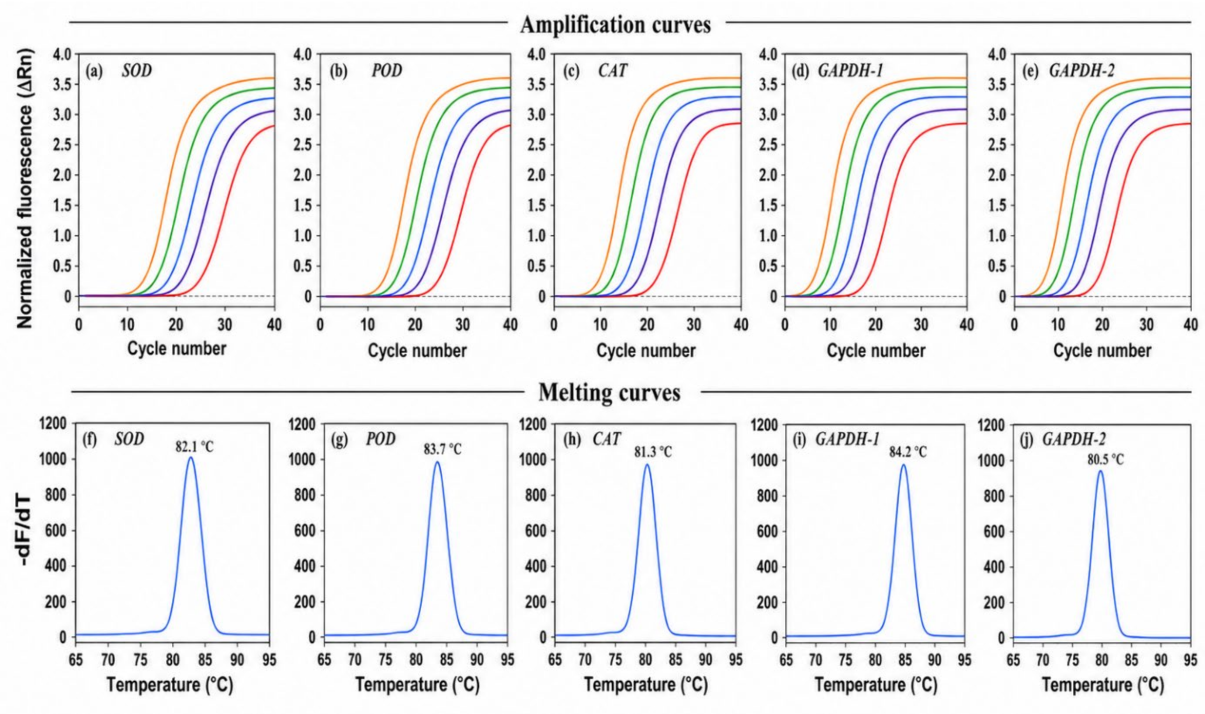

Figure S1. qRT-PCR amplification curves (SOD, POD, CAT, GAPDH-1, GAPDH-2) showing sigmoidal amplification profiles, and melting curves confirming single specific peaks for all genes in *Wedelia trilobata*. Data indicate efficient amplification and primer specificity.

Table S1. Physicochemical characteristics of rice straw biochar produced at 450 °C (RBC450) and 700 °C (RBC700).

		**Yield (%)**	**pH**	**EC (mscm^{-1})**	**Surface area (m2g^{-1})**	**DOC (mg CL^{-1})**	**C%**	**H%**	**N%**	**S%**	**Ash (%)**	**O%**
**Biochar**	450 °C	34.20	7.52	5.42	22.14	8.32	57.85	2.62	1.38	0.39	17.64	20.12
	700 °C	26.42	9.61	3.95	195.2	2.14	64.69	1.15	1.12	0.28	25.63	7.13
